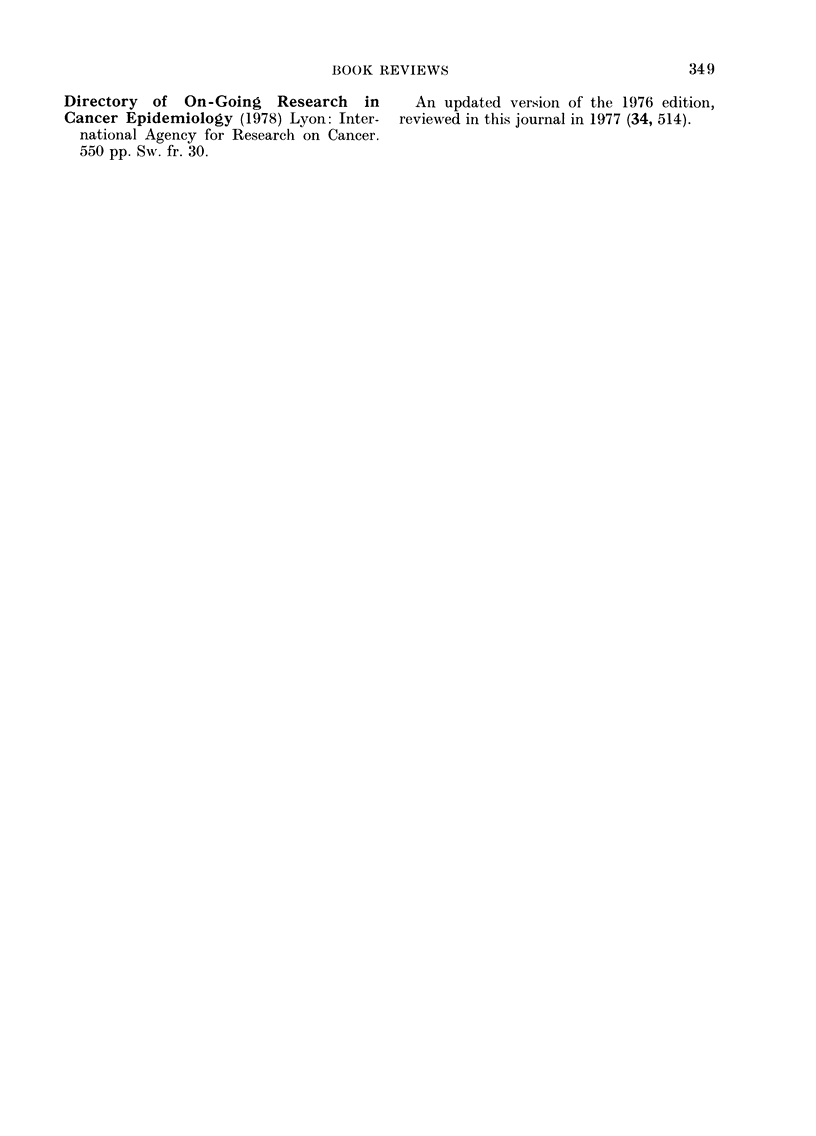# Directory of On-Going Research in Cancer Epidemiology

**Published:** 1979-03

**Authors:** 


**Directory of On-Going Research in Cancer Epidemiology** (1978) Lyon: International Agency for Research on Cancer. 550 pp. Sw. fr. 30.

An updated version of the 1976 edition, reviewed in this journal in 1977 (**34,** 514).